# Impact of sample size on optimisation algorithms for the MLP used in the prediction of client subscription to a term deposit

**DOI:** 10.12688/f1000research.168092.1

**Published:** 2025-12-22

**Authors:** Tshegofatso Botlhoko, Tlhalitshi Volition Montshiwa

**Affiliations:** 1 Department of Business Statistics & Operations Research, North West University Faculty of Economic and Management Sciences, Potchefstroom, North West, 2735, South Africa

**Keywords:** Multilayer Perceptron (MLP); Genetic Algorithm (GA); Grasshopper Optimization Algorithm (GOA); Covariance Matrix Adaptation Evolution Strategy (CMA-ES); Machine Learning; Term Deposit Subscription.

## Abstract

**Background:**

One of the disadvantages of the multilayer perception (MLP), which is a machine learning (ML) algorithm used in various fields, includes the uncontrollable growth of the number of total parameters, which may make MLP redundant in such high dimensions, and the uncontrollable growing stack of layers that ignores spatial information. Optimization algorithms were developed to determine the optimum number of parameters for MLP.

**Methods:**

In this paper, the performances of the Genetic Algorithm (GA), Grasshopper Optimization Algorithm (GOA), and Covariance Matrix Adaptation Evolution Strategy (CMA-ES) are compared. The study also sought to determine the impact of sample size variations on these optimization algorithms. A dataset on the direct marketing campaigns of a Portuguese banking institution from the UCI Machine Learning Repository with a sample size of 4 521 was used. Synthetic Minority Oversampling Technique (SMOTE) was applied to balance the binary dependent variables for the training data across various sample sizes.

**Results:**

Based on the classification accuracy, specificity, sensitivity, precision, F-score, and execution time, the MLP based on CMA-ES (CMA-ES-MLP) was identified as the best classifier overall, as it maintained high rates of these classification metrics and was the second fastest to train. CMA-ES-MLP with a training sample of 5 114 was our ideal classifier, and it competes well with the classifiers that have been built by previous studies that used the same dataset.

**Conclusions:**

The study found no consistent increase or decrease in the classification performance of the algorithms as the sample size increased, and the metrics fluctuated rapidly across sample sizes. It is recommended that future studies be conducted to compare the best-performing classifiers identified in previous studies with the CMA-ES-MLP in this study under the same experimental conditions.

## 1.1 Introduction

The multidisciplinary field of data mining includes Information Technology (IT), Artificial Intelligence (AI), Machine Learning (ML), statistics, pattern recognition, data retrieval, Neural Networks (NN), and information-based systems. ^1^ This study focused on ML classification algorithms and classifiers. A classifier is an algorithm that links input data to a specific category. ^2^ More specifically, this study focuses on the Multilayer Perceptron (MLP) classifier because it is one of the most-used algorithms in data science and in recent studies ^3– 7^ because of its flexibility and ability to differentiate data that can be split linearly ^8^ defined the MLP as a feedforward artificial neural network (ANN) that comprises the input layer, at least one hidden layer, and the output layer, which are connected by nodes. MLP is also of interest in this study because it is applicable to various fields such as speech recognition, image recognition, text classification, and machine translation software.

Although it is applicable across various disciplines, a disadvantage of MLP is that the number of total parameters in it can grow uncontrollably, whereby the number of perceptrons in layer one is multiplied by the number of parameters in layer two, which is then multiplied by the number of parameters in layer three and so on. This is inefficient because of redundancy in such high dimensions. In addition, ( ^9^: 400) stated that when flattened vectors are used as inputs, this uncontrollably growing stack of layers ignores the spatial information. These multiplying parameters can be difficult to control; hence, optimization algorithms were established to determine the optimum number of parameters for the MLP.

Reference [10] defined an algorithm as a process or equation that solves a problem by following a predetermined set of steps. Reference [11] described optimization techniques as analytical approaches that use differential calculus to find the best solution. Reference [12] further explained that the purpose of optimization techniques is mainly to handle problems that cannot be handled by classifiers. These problems consist of functions with a single variable, functions with multiple variables and no constraints, and functions with multiple variables with both equality and inequality constraints. A variety of optimization algorithms have been developed, and because of their adaptable and flexible searching processes, they have demonstrated a great degree of promise in solving optimization issues. In addition, ^13^ mentioned their capacity to use specific statistical tools to display satisfactory performance on MLP classification methods, as well as their efficiency in resolving linear and non-linear problems by avoiding local optima and balancing the exploration and exploitation trends.

According to, ^14^ there are several optimization algorithms used in optimizing the MLP, including the Bayesian optimization algorithms (BOA), binary particle swarm optimization (BPSO), Covariance Matrix Adaptation Evolution Strategy (CMA-ES), Differential Evolution (DE), FireFly Algorithm (FFA), genetic algorithms (GA), grasshopper optimization algorithm (GOA), and particle swarm optimization (PSO). Other optimization algorithms include the hybrid meta-heuristic approach, which was used in the study by, ^15, 16^ and it has been compared to other newly developed optimization algorithms that were used to form hybrid MLP models such as the Gloworm Swarm Optimization-MLP (GSO-MLP), Biogeographical-Based Optimization-MLP (BBO-MLP), and Genetic Algorithm-MLP (GA-MLP).

The scope of this study is limited to the Genetic Algorithm (GA), grasshopper optimization algorithm (GOA), and Covariance Matrix Adaptation Evolution Strategy (CMA-ES). This is because the literature comparing these novel evolutionary optimization algorithms is scarce. Therefore, although they are known to be better performers than older algorithms, the best optimization algorithm for the MLP between GA, GOA, and CMA-ES remains unknown. It is imperative to determine the most efficient optimization algorithm for an optimal MLP because each optimization technique has various reliability, strength, efficiency, utilization, and limitations. According to, ^17^ one of the disadvantages of not knowing the most efficient optimization algorithm is that it cannot determine the best level of local optima. It can also waste time for end-users of MLPs (i.e., non-statisticians/non-data scientists) to compare the optimization algorithms before fitting their MLPs, as opposed to having a study such as the current study to refer to, which has already compared these algorithms and has recommended the most efficient one(s).

This study also intended to explore the effect of changes in sample size on the efficiency of GA, GOA, and CMA-ES. This is because the increase in the sample size is known by some studies to be able to improve the accuracy and robustness of many statistical methods, as detailed by studies such as those conducted by ^18– 21^ highlighted that when the focus is on individualised outcome risk prediction, it has been shown that extremely large datasets might be needed for ML techniques. The authors explained that for binary outcomes, ML techniques could require more than ten times as many events for each predictor to achieve a small amount of over-fitting compared with classic modelling techniques such as logistic regression and might show instability and high optimism ^21^ explained that when dealing with optimization algorithms and sample size, it is vital to ensure accurate predictions in key subgroups and to consider the accurate sample size when using an existing dataset to avoid overfitting. On the other hand, although some studies advocate for a large dataset for ML algorithms, ^22^ explained that a study with a sample size that is too small has a higher risk of missing a meaningful underlying difference, while one with a sample size that is too large may be more expensive than necessary.

It is evident that sample size affects the efficiency of ML algorithms. However, the efficiency of GA, GOA, and CMA-ES when used in optimizing the MLP relative to the sample size remains unknown, and to the best of our knowledge, this has never been explored before in a single study. In this study, efficiency refers to a measure of the quality of the optimization algorithms depending on the sample size, which is evaluated using measures such as specificity, sensitivity/recall, and execution time. Therefore, this study intended to determine the impact of sample size on the efficiency of GA, GOA, and CMA-ES when used for optimizing the MLP, with a focus on these due to their wide application in various studies ^23– 27^ and because of their known effectiveness and flexibility.

A comparison of GA, GOA, and CMA-ES in optimizing MLP and the effect of sample size on the performance of these algorithms is the main objective of this study. However, as an area of application, these methods are applied to predict the likelihood of subscribing to a term deposit following telephone-based direct marketing by a banking institution. This has been the focus of application of ML classifiers in several previous studies, including. ^28– 30^ Therefore, this study intends to extend the literature in this area, which has caught the attention of many researchers when comparing the performance of ML classifiers. More details on the ML classifiers applied and the conclusions reached from these previous studies are detailed in Table 2 in the dataset section of this paper.

### 1.2 Related works on evaluation of optimisation algorithms for the MLP

Several previous studies that explored the efficiency of various optimization algorithms for MLP in different areas of application showed that the most efficient optimized MLP varies depending on the area of application, sample sizes, and evaluation metrics implemented in such studies. From the studies reviewed, the most common area of research is information technology ^31– 34^ followed by the medical sector. ^35– 37^ To extend the study by, ^38^ who focused on the financial sector, the current study uses a financial dataset, but it includes the CMA-ES-MLP and GOA-MLP, which are compared to the basic MLP and GA-MLP, which were also included in the study by, ^38^ but across different sample sizes rather than only one. From the studies reviewed, the sample sizes ranged from 400 to 8367, but only one sample was used per study. As such, the current study expands the scope of these studies by comparing the basic MLP and its optimized variates using different samples to determine the effect of sample size on the performance of these ML algorithms.

The literature shows that in all the studies, the optimized versions of the MLPs were selected as the best performers, and not the basic MLP, which was not optimized. This is evident from the studies conducted by ^38^ in which the diversity-considered GA-MLP ensemble algorithm (DGAMLPE) outperformed the unoptimized basic MLP, ^35^ in which DGAMLPE outperformed the basic MLP, and, ^31^ in which the GOA-MLP outperformed the basic MLP. “This implies that indeed the optimised variates of the MLP can improve the basic MLP, and it is also seen that optimisation algorithms give the MLP a competitive advantage over other ML classifiers such as Random Forest (RF), Extreme Gradient Boost (X-GBoost), Weighted Count of Errors and Correct (WCEC), and Deep Belief Network-Support Vector Machine (DBN-SVM), Logistic Regression (LR), K-Nearest Neighbors (K-NN), Decision Tree Classifier (DTC), Support Vector Machine (SVM), Random Forest Classifier (RFC), and Ensemble” ^35^:314). Considering these findings from the literature, the researchers were interested in optimization algorithms for the MLP in the current study. To extend the literature, the researchers included the CMA-ES-MLP in the competing models and explored the effect of sample size on these ML algorithms.

The most used optimized variates of the MLP from previous studies are GA-MLP, ^16, 32, 34, 35, 37^ followed by PSO-MLP, ^16, 31, 32, 34, 39^ and GOA-MLP, ^31, 33, 39^ but none of these studies included CMA-ES-MLP, which implies that the performance of CMA-ES-MLP against GA-MLP and GOA-MLP remains an area that requires further research. This study bridges this gap. It also appears that the most frequently used accuracy metric from the reviewed studies is classification accuracy, ^16, 31– 39^ followed by the F-measure. ^33, 35– 38^ The negative and positive predictive values appear to be the least used accuracy metrics. ^31, 35^ Other classification evaluation metrics used in previous studies included sensitivity/recall, specificity, and precision. Similarly, the current study also implemented the classification accuracy, precision, sensitivity/recall, specificity, F-measure, and execution time to compare optimized algorithms based on the popularity of these metrics in previous studies. Including a variety of comparison metrics in a single study assists in minimizing the model selection bias that may be experienced when very few similar metrics are used in the comparison and selection of the most efficient model.

## 1.3 Method

### 1.3.1 Dataset

The data used in this study is a secondary dataset on the direct marketing campaigns of a Portuguese banking institution. The dataset was obtained from the UCI Machine Learning Repository of the Center for Machine Learning and Intelligent Systems. The primary contributor to the data is. ^28^ The dataset can be accessed at https://archive.ics.uci.edu/ml/datasets/Bank+Marketing. The dataset has a total of 4 521 observations, and 11 variables were selected for use as attributes (see Table 1) in this study to predict whether a client will subscribe to a term deposit following the marketing campaign. That is, the binary variable “has the client subscribed to a term deposit” from the dataset is used as a dependent variable (binary; 0 is no and 1 is yes).

**Table 1.** Description of features.

Name of variables	Description of variable	Variable type/category
Age	Client’s age	Numeric
Type of Job	The type of job of client	Admin, blue collar, entrepreneur, housemaid, management, retired, self-employed, services, student, technician, unemployed, and unknown
Marital Status	What is the marital status of the client?	Divorced, married, single, unknown
Educational level	Highest qualification of client	Basic 4y, basic 6y, basic 9y, high school, illiterate, professional course, university degree, and unknown
Default	Does the client have credit in default?	No, yes, and unknown
Housing	Does the client have housing loan?	No, yes, and unknown
Loan	Does the client have a personal loan?	No, yes, and unknown
Contact	Contact communication type	Cellular and telephone
Day	The last contact day of the week	Monday, Tuesday, Wednesday, Thursday, Friday
Duration	Last contact duration, in seconds (numeric)	e.g., if duration = 0 then y = 'no'
Outcome of Previous Marketing Campaign		Failure, non-existent, and success

To mimic different sample sizes which are needed to study the impact of sample size on the efficiency of the MLP optimisation algorithms, nine (9) random samples of different sizes (varying by 10%) were drawn with replacement from the 4521. Samples were randomly selected at 10% difference using stratified sampling, in which the dependent variable was used as the stratum to ensure that the samples maintained the distribution of the main dataset in the dependent variable. The following random sample sizes were created: 10% (n = 452), 20% (n = 904), 30% (n = 1356), 40% (n = 1808), 50% (n = 2261), 60% (n = 2713), 70% (n = 3165), 80% (n = 3617), 90% (n = 4069), and the entire dataset, which contained 100% of the observations (n = 4521). The variables described in Table 1 were used as independent variables or features.

All categorical features with at least three (3) classes from Table 1 were converted to dummy variables using the one-hot encoding technique, which converts classes of the categorical variable to a vector that contains 1 and 0, denoting the presence and absence of the feature, respectively, which led to an increase in the number of features used in the paper to 42. Previous studies that have been conducted that focused on the application and/or comparison of ML classifiers (including MLP and its variates) on the dataset chosen for this study are summarized in Table 2.

**Table 2.** A summary of studies on the comparison and application of ML classifiers on using the dataset on direct marketing campaigns of a Portuguese banking institution from the UCI repository.

Authors	Classifiers compared or applied	Best model
Moro et al. (2014)	LR, DT, NN, and SVM.	**NN** with an Area under the area of the receiver operating characteristic curve (AUC) of 0.8 and area of the LIFT cumulative curve (ALIFT) of 0.7
Ghatasheh et al. (2020)	Meta-Cost-MLP, Cost Sensitive Classifier-MLP, MLP (Baseline), DL-MLP, J48, LL, DT, Very Fast Decision Rules (VFDR) and RF.	**Meta-cost MLP** with recall of 0.808, precision of 0.771, Geometric mean of 78.93%, and Classification accuracy of 77.48%.
Moro et al. (2011)	NB, DT and SVM.	**SVM** with AUC of 0.938 and ALIFT=0.887.
Asare-Frempong and Jayabalan (2017)	MLP, DT (C4.5), LR and RF	**RF** with classification accuracy of 86.08% and AUC of 92.7%.
Moro et al. (2015) ^42^	Customer lifetime value (LTV) based NN (LTV-NN), baseline NN (with no historical data),	**LTV-NN ** increased the AUC of the baseline-NN from 0.8002 to 0.8609, while ALIFT improved from 0.6701 to 0.7044 where AUC was at least 0.84, and ALIFT was at least 0.69.
Elsalamony (2014)	MLP, NB, LR, and the Ross Quinlan new DT (C5.0).	Based on the testing dataset, MLP produced the highest classification accuracy of 90.49%, LR the highest sensitivity of 65.53%, and C5.0 yielded specificity of 93.23%.
Zaki et al. (2024)	Stochastic Gradient Descent (SGD) Classifier, k-nearest neighbour Classifier, and Random Forest Classifier.	**DT** with a classification accuracy of 87.5%, a negative predictive value (NPV) of 93%, and a positive predictive value (PPV) of 87.8%.
Ładyżyński et al. (2019) ^44^	RF, classification and regression tree (CART) and deep belief learning implemented in H2O framework, and deep belief networks implemented in H2O framework with l1 regularization parameter added.	**CART** with a precision of 9.01% and recall of 67.27%, and the authors commented it is the most efficient in terms of computing power.
Pavlović et al. (2014) ^45^	DT	**DT** yielded classification accuracy of 88.51%, sensitivity of 93.6%, specificity of 50.1%, AUC of 70.5%, and Brier of 20.5%.
Karim and Rahman (2013) ^46^	NB and DT (4.5).	**DT (C4.5)** with classification accuracy of 94%, precision for “yes” of 79.1%, precision for “no” of 95.5% and AUC of 93.3, but the DT (C4.5) was 5.78 seconds slower to train than the NB.
Kim and Street (2004)	Baseline ANN and Genetic Algorithm (GA) based ANN (GA-ANN).	**GA-ANN **.

Table 2 shows that 2004 to date, several ML classifiers have been evaluated to predict the likelihood of a client to subscribe to a term loan following a direct marketing campaign by the bank using data from a Portuguese banking institution. In general, the table shows that the results vary depending on the setting such as the number of attributes, the number of observations in the data, and the number of training times to mention a few. Most of these studies included neural networks ^29, 40– 43, 47^ including the basic MLP and its variates such as Meta-Cost-MLP, Cost sensitive classifier-MLP, and the GA based ANN (GA-ANN). Although it appears in most previous studies, the basic neural networks classifier was only found to be the best performer when compared to LR and DT and SVM in the study by. ^28^ However, whenever its modified variates were included in the comparison, these variates were found to be best performers against the basic MLP such as in the study by ^29^ in which the Meta-Cost-MLP outperformed the basic MLP and other classifiers such as (J48, LL, DT, VFDR), and in the study by ^47^ in which the GA-ANN outperformed the baseline ANN. These results show that making improves to the basic MLP can improve its performance, hence this paper extend literature around the enhancement of the neural networks (specifically the MLP) as done by some authors in Table 2, by comparing GA, GOA and CMA-ES optimisation algorithms for the MLP using the direct marketing data used in studies that are summarise in this table. It is evident from Table 2 that these optimisation algorithms have never been compared in a single study using the dataset that was used by the studies in Table 2.

### 1.4 Data analysis methods

**1.4.1 Data balancing**

The data in this study were split into 80% training data and 20% testing data, which is a commonly used train-to-testing data-splitting ratio. A Synthetic Minority Oversampling Technique (SMOTE) was used to balance the training samples ^48^ defined SMOTE as one of the most used oversampling techniques to solve imbalanced data problems, and it aims to balance class distributions by randomly increasing minority class examples by replicating them ^48^ explained that SMOTE uses linear interpolation to generate the virtual training records. These synthetic data were generated through a random selection of at least one *k*-nearest neighbor for each observation in the minority class. ^48^ In this study, SMOTE was chosen because of its advantage in reducing the risk of overfitting and its wide application in many previous studies, such as. ^48– 52^

**Figure 1.**
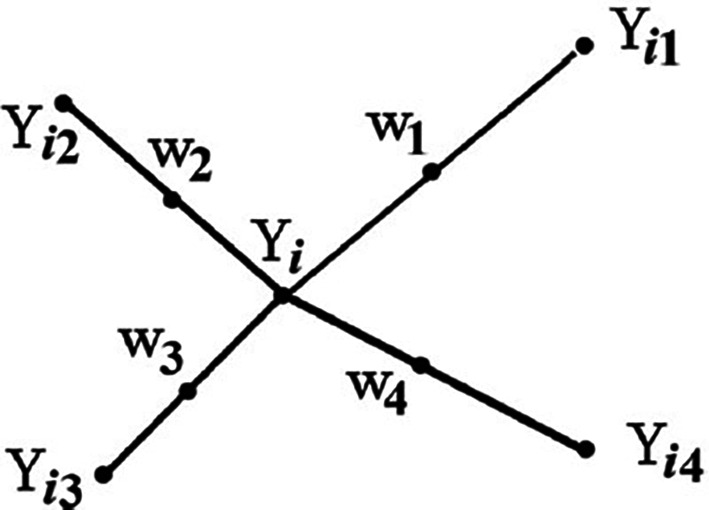
Example of how to generate synthetic data using SMOTE ( ^53^:1414). In Figure 1 explains how SMOTE randomly generates synthetic data ( $w_{1}$ to $w_{4}$ ) to balance the imbalanced dataset by taking the difference between the nearest neighbours ( $Y_{i1}$ to $Y_{i4}$ ) of the data point under consideration ( $Y_{i})\;$ and multiplying $Y_{i}$ by a random number between 0 and 1, and then adding it to the feature vector under consideration. ^53^

From Figure 1, $Y_{i}$ is the point under consideration, $Y_{i}1$ to $Y_{i}4$ are the nearest neighbors, and $w_{1}$ to $w_{4}$ represent the synthetic data generated by the randomized interjection ^53^ explained that synthetic samples are generated by considering the difference between the nearest neighbor and the feature vector ^53^ further explained that the difference is multiplied by a random number between 1 and 0 and then added to the feature vector under consideration. Table 3 presents balanced training data from the original dataset.

**Table 3.** Frequencies of the dependent variable in the SMOTE balanced training sets across the sample sizes.

Unbalanced data				Balanced data			
Sample Size	Client Subscription	N	%	Sample Size	Client Subscription	N	%
n = 362	Unsubscribed	313	86	n = 626	Unsubscribed	313	50
	Subscribed	49	14		Subscribed	313	50
n = 723	Unsubscribed	640	89	n = 1280	Unsubscribed	640	50
	Subscribed	83	11		Subscribed	640	50
n = 1085	Unsubscribed	970	89	n = 1940	Unsubscribed	970	50
	Subscribed	115	11		Subscribed	970	50
n = 1446	Unsubscribed	1296	90	n = 2592	Unsubscribed	1296	50
	Subscribed	150	10		Subscribed	1296	50
n = 1809	Unsubscribed	1602	89	n = 3024	Unsubscribed	1602	50
	Subscribed	207	11		Subscribed	1602	50
n = 2170	Unsubscribed	1916	88	n = 3832	Unsubscribed	1916	50
	Subscribed	254	12		Subscribed	1916	50
n = 2026	Unsubscribed	1791	88	n = 3582	Unsubscribed	1791	50
	Subscribed	235	12		Subscribed	1791	50
n = 2894	Unsubscribed	2557	88	n = 5114	Unsubscribed	2557	50
	Subscribed	337	12		Subscribed	2557	50
n = 3255	Unsubscribed	2880	88	n = 5760	Unsubscribed	2880	50
	Subscribed	375	12		Subscribed	2880	50
n = 3617	Unsubscribed	3199	88	n = 6398	Unsubscribed	3199	50
	Subscribed	418	12		Subscribed	3199	50

Table 3 shows that the class of the dependent variable is balanced after using SMOTE specifically for the training data samples. In all the samples, equal numbers of unsubscribed participants and subscribed participants are observed.

**1.4.2 Multilayer Perceptron (MLP)**

Explained that MLP was invented in 1958 at the Cornell Aeronautical Laboratory by Frank Rosenblatt, funded by the Office of Naval Research in the United States. ^54^ Further explained that although it was originally designed as a machine rather than a program, the perceptron was first implemented in IBM 704 as software before being implemented in specially designed hardware as the “Mark 1 perceptron.” In addition, ^55^ explained that the purpose of this machine is image recognition; it has 400 photocells arranged in an array and randomly connected to the “neurons.” According to the author, electric motors update the weights during learning, and the weights are encoded in the potentiometers. The flexibility of the MLP has enabled its function in various activities. ^56^ It has only been used for image recognition, ^57, 58^ speech recognition, ^59^ and machine translation software. ^60^ Currently, it can be used for text data, ^61, 62^ speech recognition, ^58^ and other types of data. MLP can be fitted using various software, such as Waikato Environment for Knowledge Analysis 3.9 (WEKA), Statistical Package for the Social Sciences (SPSS), and Python. With the use of optimization algorithms, such as those being compared in this study, MLPs have become very useful, convenient, and easy to use.

The MLP consists of an input and an output layer with one or more hidden layers of non-linear activating nodes. ^63^ Each node in one layer connects with a certain weight to every node in the following layer. ^63^ In the input layer, the activations, which were defined by ^64^ as the source of the MLP’s power, were determined using the following equation:

$$b_{j}=\sum_{i=0}^{D}w_{\mathrm{ij}}^{\left(1\right)}x_{i},$$ (1)

The first layer involves M linear combinations of the d-dimensional input for

i1,2,…,Mandj=1,2,…,d,

where $w_{\mathrm{ij}}^{\left(1\right)}$ are the weights for node *j* in layer 1 for incoming node *I* and (1) indicates that this is the first layer of the network. Each activation was then transformed by a non-linear activation function *g.*

In this study, tanh was used as the activation function for the hidden layer ^65^ described the Tanh function as a smoother, zero-center function, with a range between -1 and 1. The Tanh function is defined by the following equation sourced from ^65^:

$$f\left(x\right)=\left(\frac{e^{x}-e^{-x}}{e^{x}+e^{-x}}\right),$$ (2)

where x is an input to the neuron and e is Euler’s number.

A sigmoid function was used as the activation function for the output layer. ^65^ defined the sigmoid as a non-linear activation used mostly in feedforward neutral networks. “It is a bounded differentiable real function, defined for real input values, with positive derivatives everywhere and some degree of smoothness” ( ^65^:5). The sigmoid activation function is given by the following relationship, sourced from ^65^:

$$f\left(x\right)=\frac{1}{1+\exp\left(-b_{j}\right)}$$ (3)

where f(x) corresponds to the outputs of the basis functions and is interpreted as the output of the hidden units.

**1.4.3 Covariance Matrix Adaptation Evolution Strategy (CMA-ES)**

The Covariance Matrix Adaptation Evolution Strategy (CMA-ES) was developed by Hansen et al. in 2003. ^66^ According to, ^67^ the algorithm’s theoretical underpinnings include variable metrics, and the CMA-ES uses maximum-likelihood updates in conjunction with a stochastic variable-metric approach. In an algorithm that quickly converges to the global optimum across a wide class of functions, the covariance matrix maximizes likelihood while resembling an expectation-maximization algorithm ^68, 69^ explained that the CMA-ES algorithm has certain drawbacks, such as its performance becoming slow if the number of model parameters that need to be estimated is large. The approximation of gradients without assuming or requiring their existence is another flaw of this algorithm. CMA-ES is a plausible candidate for an effective parameter estimation algorithm, ^70^ but it must be tested against other algorithms to ascertain its efficiency, particularly when the sample size is varied.

The CMA-ES samples from the multivariate normal distribution search rank the sampled points according to their fitness function values. The multivariate normal distribution can be calculated using the following equation obtained by ^71^:

$$x_{i}∼N\left(m_{k},\sigma_{k}^{2}C_{k}\right),$$ (4)

$$∼m_{k}+\sigma_{k}\times N\left(0,C_{k}\right),$$ (5)

where $m_{k}$ is the distribution average and recent favorite solution to the optimization problem, $\sigma_{k}$ is the step size, and $C_{k}$ is the symmetric and positive definite

The fitness function for the CMA-ES is defined as:

$$f\left(x\right)=g\left(x^{T}\mathrm{Hx}\right),$$ (6)

where Hx is the Hessian matrix of f(x) and $x^{T}$ is the transpose of x .

The mean distribution is then updated to a weighted average using the following equation:

$$m_{\mathrm{new}}\leftarrow \sum_{i=1}^{\mu }w_{i}x_{i:\lambda }=m+\sum_{\;i=1}^{\mu }\mathrm{wi}\left(x_{i:\lambda }-m\right),$$ (7)

where $m_{\mathrm{new}}\;$ is the new distribution mean, μ is the number of parameters, λ is the population size, m is the mean vector, and $w_{i}$ is the recombination weight.

The isotropic evolution is then updated using the following equation:

$$p_{\sigma }\leftarrow \left(1-C_{\sigma }\right)p_{\sigma }+\sqrt{1-{\left(1-C_{\sigma }\right)}^{2}}\sqrt{\mu_{w}}C_{k}^{\frac{1}{2}}\frac{m_{k+1}-m_{k}}{\sigma_{k}},$$ (8)

where $p_{\sigma }$ is the evolution path, $\left(1-C_{\sigma }\right)$ is the discount factor, $\sqrt{1-{\left(1-C_{\sigma }\right)}^{2}}$ is the complement for the discounted variance, and $\sqrt{\mu_{w}}C_{k}^{\frac{1}{2}}\frac{m_{k+1}-m_{k}}{\sigma_{k}}$ are distributed as N(0,I) under neutral selection.

$$\sigma_{k+1}=\sigma_{k}\times \exp\left(\frac{C_{\sigma }}{d_{\sigma }}\left(\frac{\left||p_{\sigma }|\right|}{E\left||N\left(0,I\right)|\right|}-1\right)\right),$$ (9)

where $\frac{C_{\sigma }}{d_{\sigma }}\left(\frac{\left||p_{\sigma }|\right|}{E\left||N\left(0,I\right)|\right|}-1\right)$ is unbiased about 0 under unbiased selection.

$$E\left||N\left(0,I\right)|\right|=\sqrt{2}\left(\frac{n+1}{2}\right)\left(\frac{n}{2}\right),$$ (10)

$$\approx \sqrt{n}\left(1-\frac{1}{4n}+\frac{1}{21n^{2}}\right),$$ (11)

Update of the covariance matrix adopted is described as follows:

$$p_{c}\leftarrow \left(1-C_{c}\right)p_{c}+1_{\left(0,\alpha \sqrt{n}\right)}\left(||p_{\sigma }||\right)\sqrt{1-{\left(1-c_{c}\right)}^{2}}\sqrt{\mu_{w}}\;\frac{m_{k+1}-m_{k}}{\sigma_{k}},$$ (12)

The CME-ES is finally updated using:

$$C_{k+1}=\left(1-c_{1}-c_{\mu }+c_{s}\right)C_{k}+c_{1}p_{c}p_{c}^{T}+c_{\mu }\sum_{i=1}^{\mu }w_{i}\frac{x_{i:\lambda }-m_{k}}{\sigma_{k}}\;{\left(\frac{x_{i:\lambda }-m_{k}}{\sigma_{k}}\right)}^{T}$$ (13)

where $c_{s}$ is the small variance loss, $c_{1}$ is the learning rate for updating the covariance matrix, and $c_{\mu }$ is the learning rate for rank- μ for updating the covariance matrix.

**1.4.4 Genetic Algorithm (GA)**

Reference [73] proposed a learning machine called the Genetic Algorithm (GA), which paralleled the principles of evolution. Barricelli (1954) pointed out that the first computer simulation of evolution was created in 1954 at the Institute for Advanced Study in Princeton, New Jersey, thanks to the efforts of Barricelli ^73^ found that GA has some limitations, such as repeated evaluation of the fitness function and difficulties in working with dynamic datasets; it tends to converge to a local optimum or even arbitrary points, instead of the global optimum of the problem. “A better solution is only in comparison to other solutions, and the stop criterion is not clear in every problem” ( ^73^:226). On the other hand, GA has been noticed to be a very efficient and effective technique for both optimisation and ML applications. ^74^ Another advantage of GA is that it requires less information about the problem ^75, 76^ stated that GA can work very well on mixed (discrete and/or continuous) problems. “The GA can be applied in real world situations such as engineering design, to make the design cycle process fast and economical, and in robotics too, to create learning robots which will behave as humans and will do tasks like cooking and laundry” ( ^77^: 347).

The efficiency of GAs depends on mutation and crossover operators and their relationships. “To determine the most appropriate operators, different mutation and crossover operators are used and they are compared with each other since GA involves a process of complex interaction between its parameters” ^78^ suggested that for the algorithm to perform best, the population size must range between 50 and 100 observations. In this study, we verified this recommendation by studying the effectiveness of GA in different sample sizes ^79^ stated that the algorithm comprises four main steps: selection, reproduction, replacement, and termination. The steps are as follows:

*1.4.4.1 Selection*

Reference [80] explained that by choosing the reproduction of offspring, the primary goal of this phase is to identify the area with the highest likelihood of producing a solution to the problem that is superior to that of the previous generation. The authors add that the selection of individuals will then be arranged in pairs of two to enhance reproduction ^79^ also explained that individuals will then pass on their genes to the next generation. “The GA uses the fitness proportionate selection technique to ensure that useful solutions are used for recombination” ( ^79^: 3). Fitness proportion selection is defined by the author as the most popular method of parent selection, where every individual can become a parent with a probability that is proportional to its fitness. “Fitter individuals have a higher chance of mating and propagating their features to the next generation. Therefore, such a selection strategy applies a selection pressure to the more fit individuals in the population, evolving better individuals over time”( ^80^: 16). The fitness proportionate selection can be calculated using the following equation adopted from ^80^:

$$p_{i}=\frac{f_{i}}{\sum_{\;j=1}^{N}f_{i}}$$ (14)

where $\;f_{i}$ denotes the fitness of individual i in the population, N denotes the number of individuals in the population, and $p_{i}$ denotes the probability.

*1.4.4.2 Reproduction*

Reference [80] explained that the algorithm applies variation operators to the parent population during the reproduction phase, creating a child population. This phase has four main operators, crossover, mutation, replacement, and termination, which are discussed below.

*1.4.4.3 Crossover*

According to, ^81^ the crossover operator swaps the genetic information of two parents to produce offspring ^81^ also explained that this is performed on parent pairs that are selected randomly to generate a child population of equal size to the parent population. For this study, a single-point crossover was considered. “Single point crossover works in such a way that a parent organism string is selected. All data beyond this point in the organism string were swapped between the two parent organisms. Strings are characterized by positional bias” ( ^81^: 13).

*1.4.4.4 Mutation*

The mutation operator adds genetic information to the new child population. According to, ^82^ the operator achieves this by flipping some bits in the chromosome to solve the problem of local minima and enhance diversification. In the present study, a bit-flip mutation was considered. “Bit flip mutation works in such a way that it selects one or more random bits and flip them. This can only be done for binary encoded GA’s” ( ^82^: 47).

*1.4.4.5 Replacement*

Reference [80] elucidated that the replacement operator acts as the final generational step to replace the old population with the new child population. In this study, a generational replacement operator is used, where the previous generation is replaced with a newly generated child population.

*1.4.4.6 Termination*

Reference [80] explains that termination is only possible in specific situations, such as having reached an absolute number of generations but not having improved the population for X iterations or the objective function value reaching a pre-defined threshold ^79^ cited a genetic algorithm example in which a counter was maintained to record generations for which the population did not improve. “Initially, we set the counter to zero. Each time we do not generate an offspring, which is better than the individuals in the population, we increase the counter. However, if the fitness of any offspring is better, then we reset the counter to zero” ( ^79^: 2). The author also stated that the algorithm terminates when the counter reaches a predetermined value.

**1.4.5 Grasshopper Optimisation Algorithm (GOA)**

The Grasshopper Optimisation Algorithm (GOA) is a new swarm intelligence algorithm and population-based method developed by Seyedali Mirjalili in 2017. ^83^ According to the authors, the GOA mainly observes the behavior of grasshopper swarms and their social interactions. Every grasshopper in the population represents a solution, and its location within the swarm is determined by three forces: wind advection, the force of gravity applied to it, and social interactions with other grasshoppers. ^84^ The process of optimizing the grasshopper algorithm involves several steps, including initialization, creation, and evaluation of the first population, identification of the best overall solution, updating the decreasing coefficient parameter, mapping the grasshopper’s distance, and updating the solution. ^85^

Reference [87] explained that the GOA can improve the average fitness of all grasshoppers, which helps the GOA effectively increase the first randomly generated solutions. The algorithm can be computed using software such as Matrix Laboratory (MATLAB) and Python. No information relating to the GOA in comparison with other algorithms has emerged, as this is a newly developed algorithm. Therefore, little is known about the efficiency of this algorithm compared to its predecessors; hence, the proposed study seeks to expand the scope of this algorithm. Grasshopper position ( $X_{i}$ ) calculations depend on three types of forces: social interactions and other grasshoppers, wind advection, and gravitational force. ^87^ All equations used in the description of the GOA in this study were sourced from ^87^ the grasshopper’s position is defined as:

$$X_{i}=S_{i}+G_{i}+A_{i},$$ (15)

where $X_{i}\;$ defines the position of the *i*-th grasshopper, $S_{i}$ is the social interaction, $G_{i}\;$ is the gravitational force on the *i*-th grasshopper, and $A_{i}$ is wind advection.

From Equation 15, social interaction is defined as:

$$S_{i}=\sum_{J=1}^{N}s\left(d_{\mathrm{ij}}\right)\hat{d_{\mathrm{ij}},}$$ (16)

where *d _ij_* is the distance between grasshopper *i* and grasshopper *j* in the $d^{\mathrm{th}}$ dimension.

From Equation 15, the gravitational force ( $G_{i})$ on the grasshopper is computed as follows:

$$G_{i}=-g_{\hat{e_{g}}},$$ (17)

where −g denotes the gravitational constant and ${\hat{e}}_{g}$ is the unit vector towards the center of the earth.

From Equation 15, the wind advection is computed as follows:

$$A_{i}={\hat{\mathrm{ue}}}_{w},$$ (18)

where u is a constant drift and ${\hat{e}}_{g}$ represents a unity vector towards the direction of the wind.

When substituting Equations 16– 18 into Equation 15, the position of the current grasshopper becomes.

Xi=∑j=1j≠1Ns(|xj−xi|)xj−xidij−ge^g+ue^w (19)

where N is the total number of grasshoppers.

Reference [84] explained how the pseudocode of the GOA algorithm works. The GOA starts optimization by creating a set of random solutions; the search agents then update their positions, followed by the determination of the position of the best target obtained thus far, and this position is updated in each iteration. ^83^ Additionally, the distances between grasshoppers were normalized in each iteration ^83^ stated that position updating is performed iteratively until the end criterion is satisfied. Finally, the position and fitness of the best target are returned as the best approximation of the global optimum.

**1.4.6 Model comparison criteria**

Precision, sensitivity/recall, F-score, classification accuracy, sensitivity, specificity, and execution time were used to evaluate and compare the optimization algorithms for the MLP, as described in this section. The classifier with the highest precision, recall, F-score, accuracy rate, sensitivity, specificity, and lowest execution time is preferred.

Classification accuracy (also referred to as overall accuracy) was described by ^88^ as the number of correct forecasts divided by the total number of forecasts. It is the most straightforward clustering quality measure proposed by ^89^ to assess the clustering results related to the ground truth. ^88^ Classification accuracy was calculated by ^88^ as follows:

$$\text{Accuracy}=\frac{\text{True Positives}+\text{True Negatives}}{\left(\text{Positives}+\text{Negative}\right)}$$ (20)

Reference [91] characterized specificity as a proportion of the extent of real negatives that are effectively distinguished, and they described the specificity equation as follows:

$$\text{specificity}=\frac{\text{True Negatives}}{\text{True Negatives}+\text{False Positives}}$$ (21)

Precision was defined by ^91^ as a measure of how close a series of measurements are to one another. The author explained that precise measurements are highly reproducible, even if the measurements are not near the correct value. Precision was calculated as follows ^91^:

$$\text{Precision}=\frac{\text{True Positives}}{\text{True Positives}+\text{False Positives}}$$ (22)

Reference [91] characterize the sensitivity/recall rate as a measure of the proportion of real positives that are accurately identified. The following equation for recall/sensitivity was adopted from ^90^:

$$\text{Sensitivity}/\text{recall}=\frac{\text{True Positives}}{\text{True Positives}+\text{False Negatives}}$$ (23)

Reference [93] defined the F-measure as a weighted harmonic mean of recall and precision. There are several motivations for this choice ^92^ explains that the harmonic mean is commonly appropriate when averaging rates or frequencies, but there are also a set of theoretical reasons. The author further explains that the mean allows differential weighting of recall and precision, but they are commonly given equal weights. The F-measure was computed as follows:

$$F=2*\frac{\text{Precision}*\text{Recall}}{\text{Precision}+\text{Recall}}$$ (24)

Execution time is defined by ^93^ as the amount of time spent by the system executing a given task, including the amount of time it spends executing runtime or system services.

## 1.5 Results

**Figure 2.**
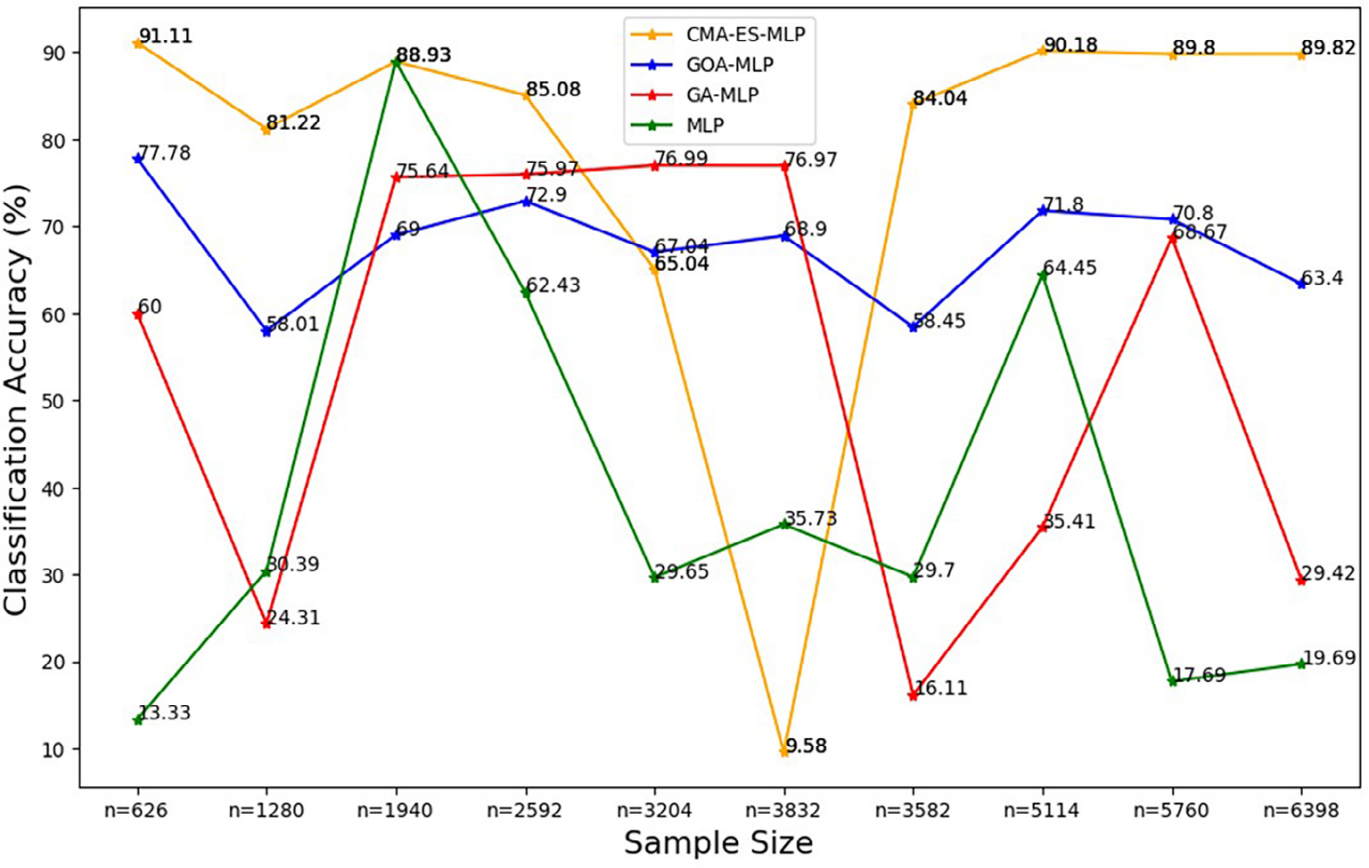
Classification accuracy for the basic MLP, GA-MLP, GOA-MLP and CMA-ES-MLP by sample size. The line graphs represent the overall classification accuracy of the basic MLP and each optimised MLP to determine the impact of various sample sizes on their classification accuracy. The classification accuracy is rate at which the model correctly classifies all the observations (both non-subscriptions and subscriptions).

**Figure 3.**
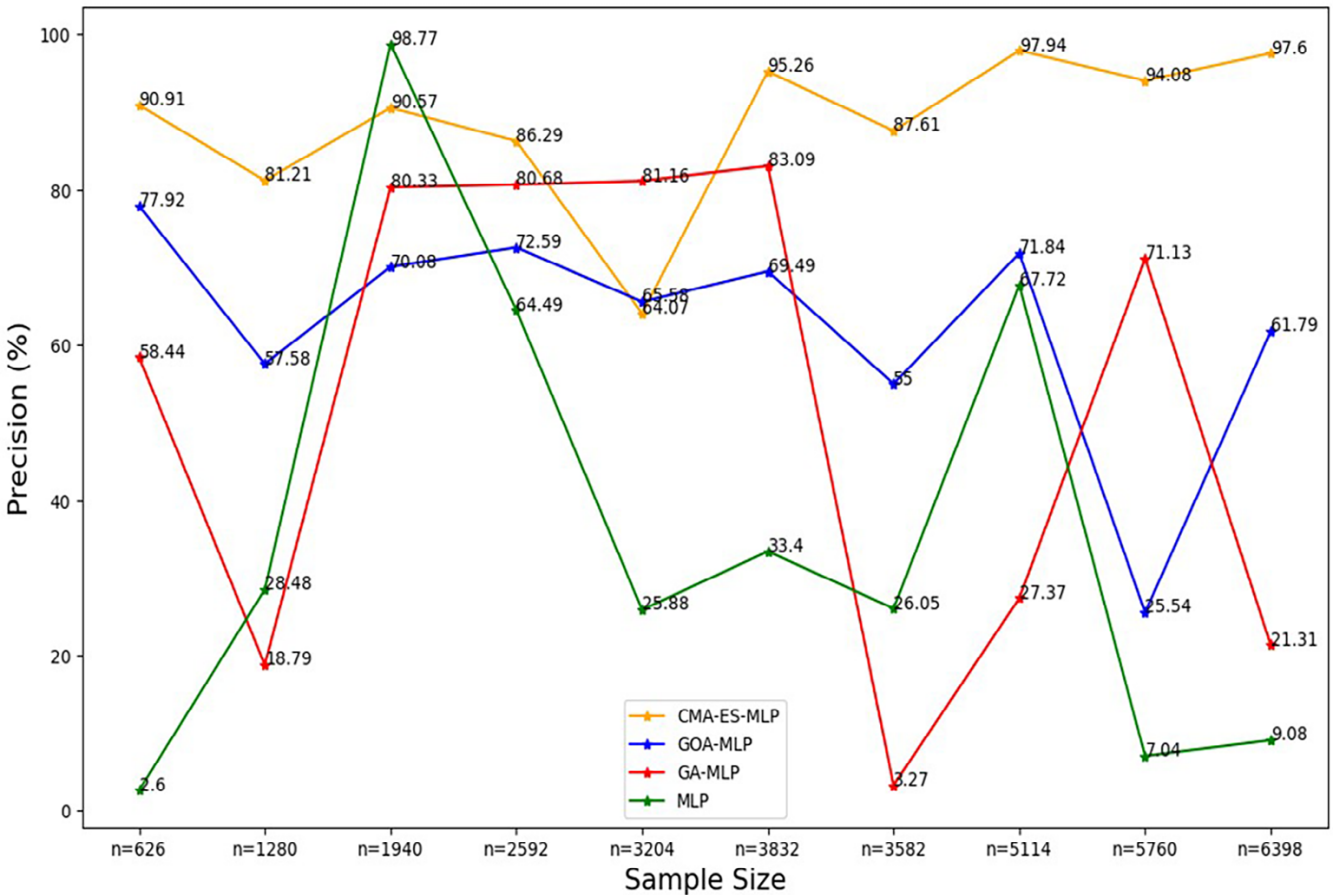
Precision rate for the basic MLP, GA-MLP, GOA-MLP and CMA-ES-MLP by sample size. The line graphs represent the overall precision rate of the basic MLP and each optimised MLP across the various sample sizes. This was to determine the impact of various sample sizes on the percentage of the term deposit subscriptions that are correctly classified by the models under comparison out of all the cases that were predicted as term deposit subscriptions by these models.

**Figure 4.**
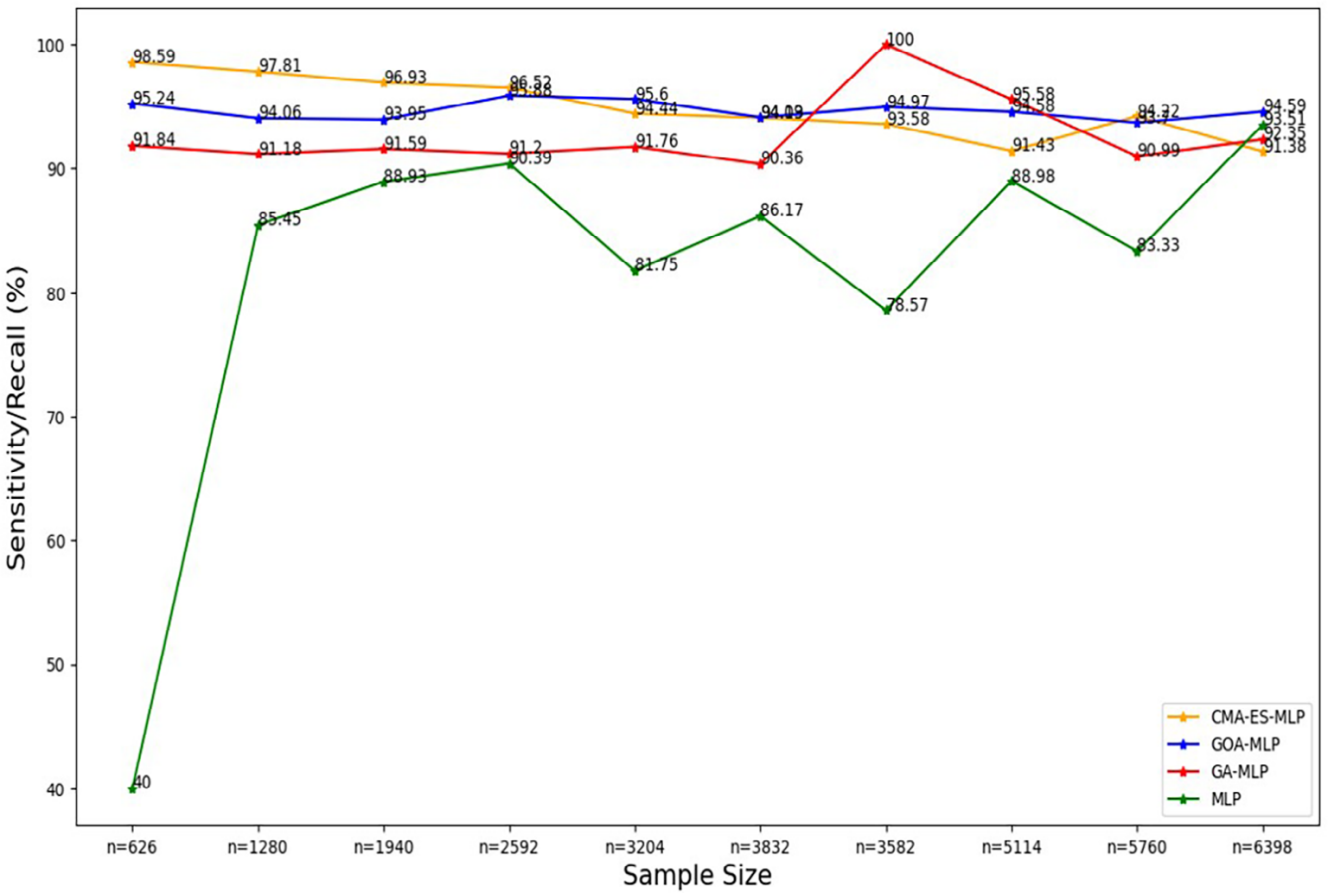
Sensitivity/recall for the basic MLP, GA-MLP, GOA-MLP and CMA-ES-MLP by sample size. The line graphs represent the sensitivity rate of the basic MLP and each optimised MLP across the various sample sizes. This was to determine the impact of various sample sizes on the percentage of the term deposit subscriptions that are correctly classified by the models under comparison out of all the term deposit subscriptions from the testing datasets.

**Figure 5.**
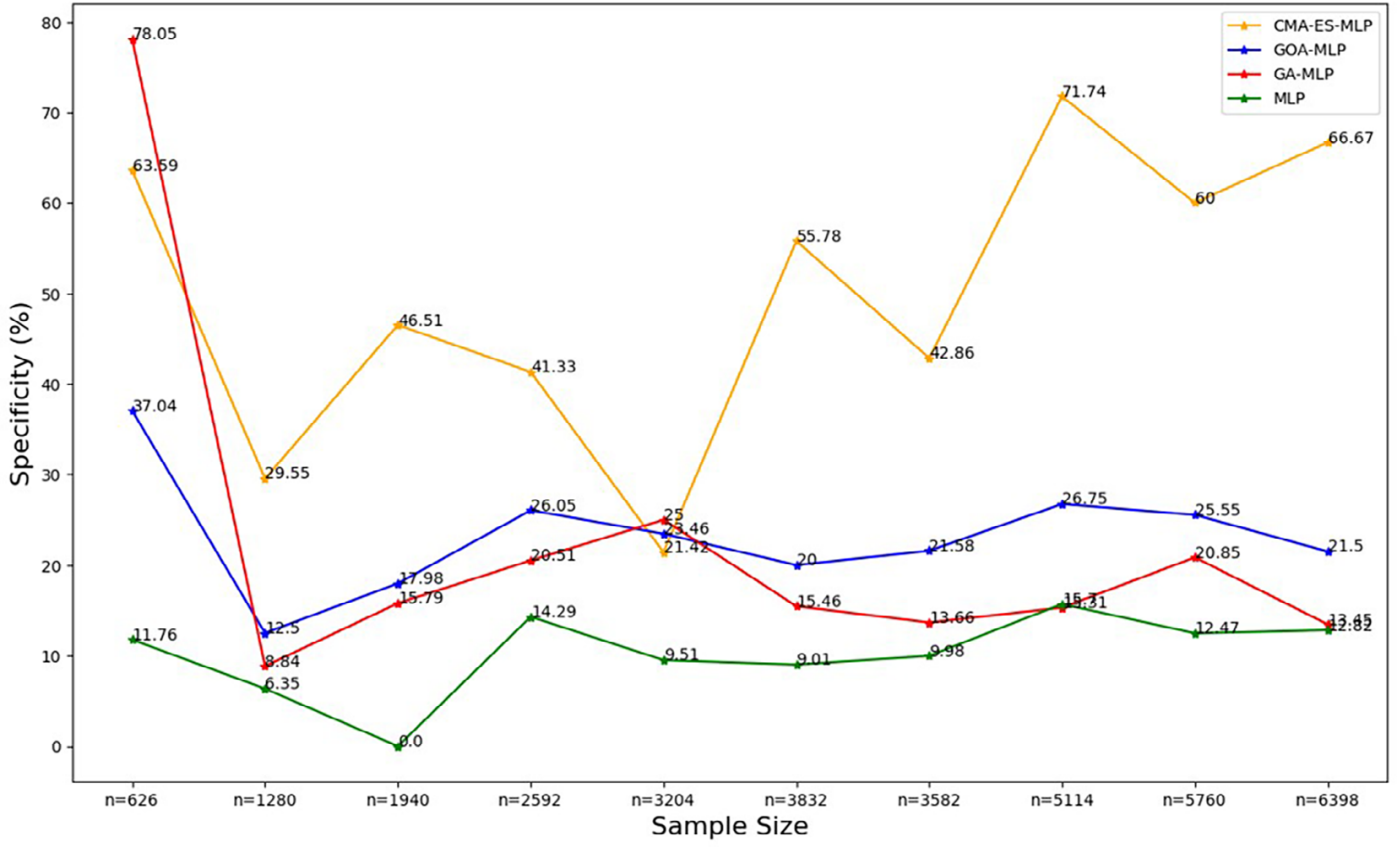
Specificity rates for the basic MLP, GA-MLP, GOA-MLP and CMA-ES-MLP by sample size. The line graphs represent the specificity rate of the basic MLP and each optimised MLP across the various sample sizes. This was to determine the impact of various sample sizes on the percentage of the term deposit non-subscriptions that are correctly classified by the models under comparison out of all the term deposit non-subscriptions from the testing datasets.

**Figure 6.**
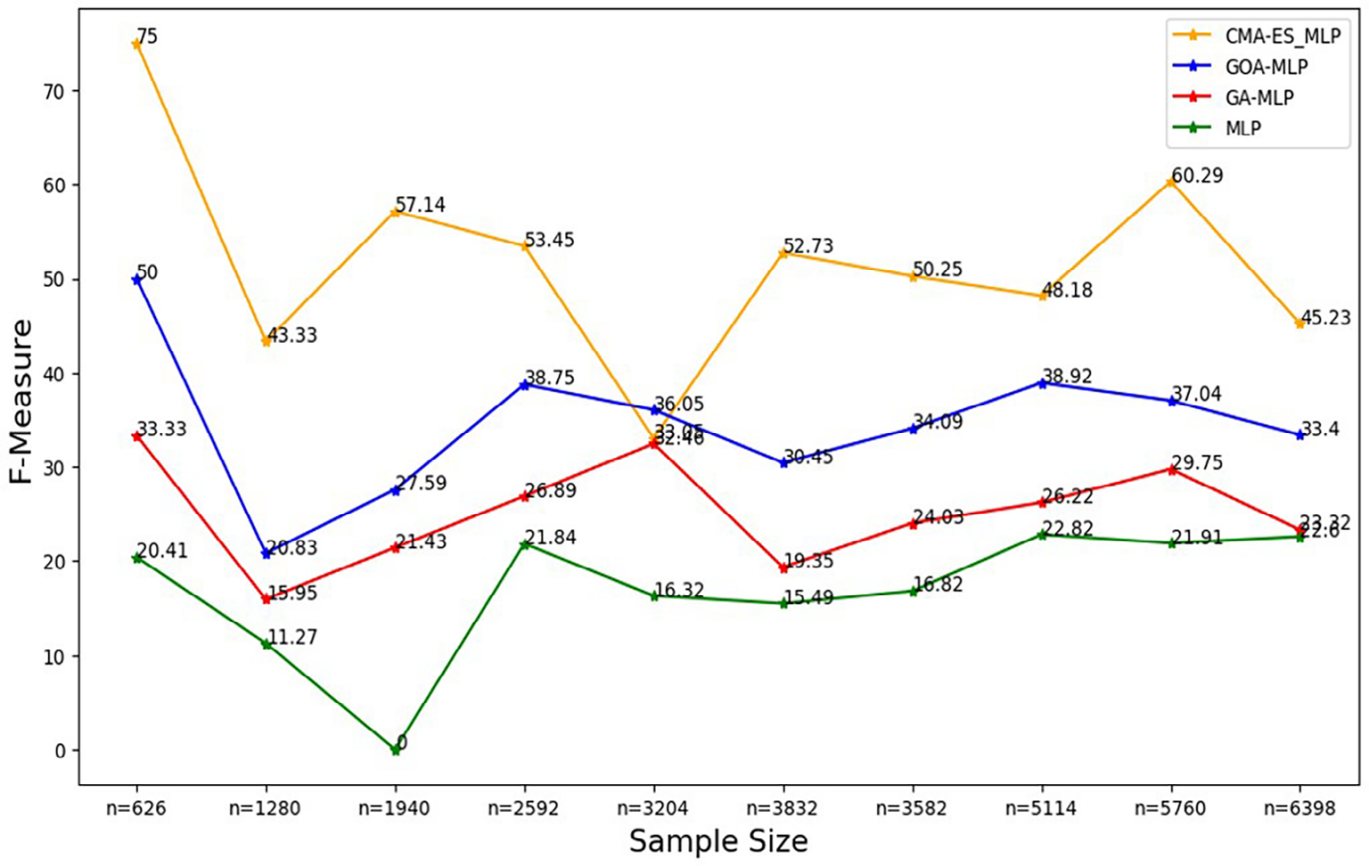
F-measure rates for the basic MLP, GA-MLP, GOA-MLP and CMA-ES-MLP by sample size. The line graphs represent the F-measure rate of the basic MLP and each optimised MLP across the various sample sizes. This was to determine the impact of various sample sizes on the harmonic mean of precision and recall. That is, how the sample size impacts the ability of the models under comparison to balance precision and recall.

**Figure 7.**
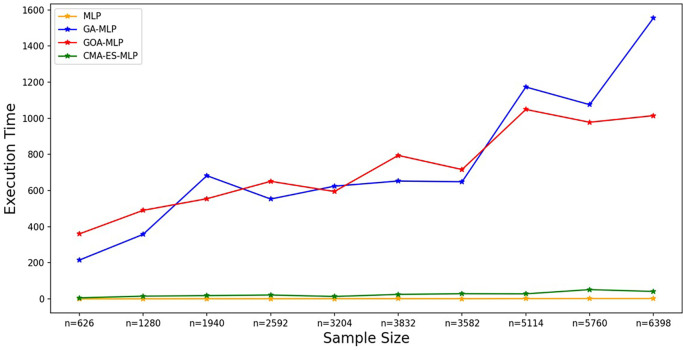
Execution times for the basic MLP, GA-MLP, GOA-MLP and CMA-ES-MLP by sample size. The line graphs represent the execution time of the basic MLP and each optimised MLP across the various sample sizes. This was to determine the impact of various sample sizes on the time it takes to complete the processes of deriving each model.

**Figure 8.**
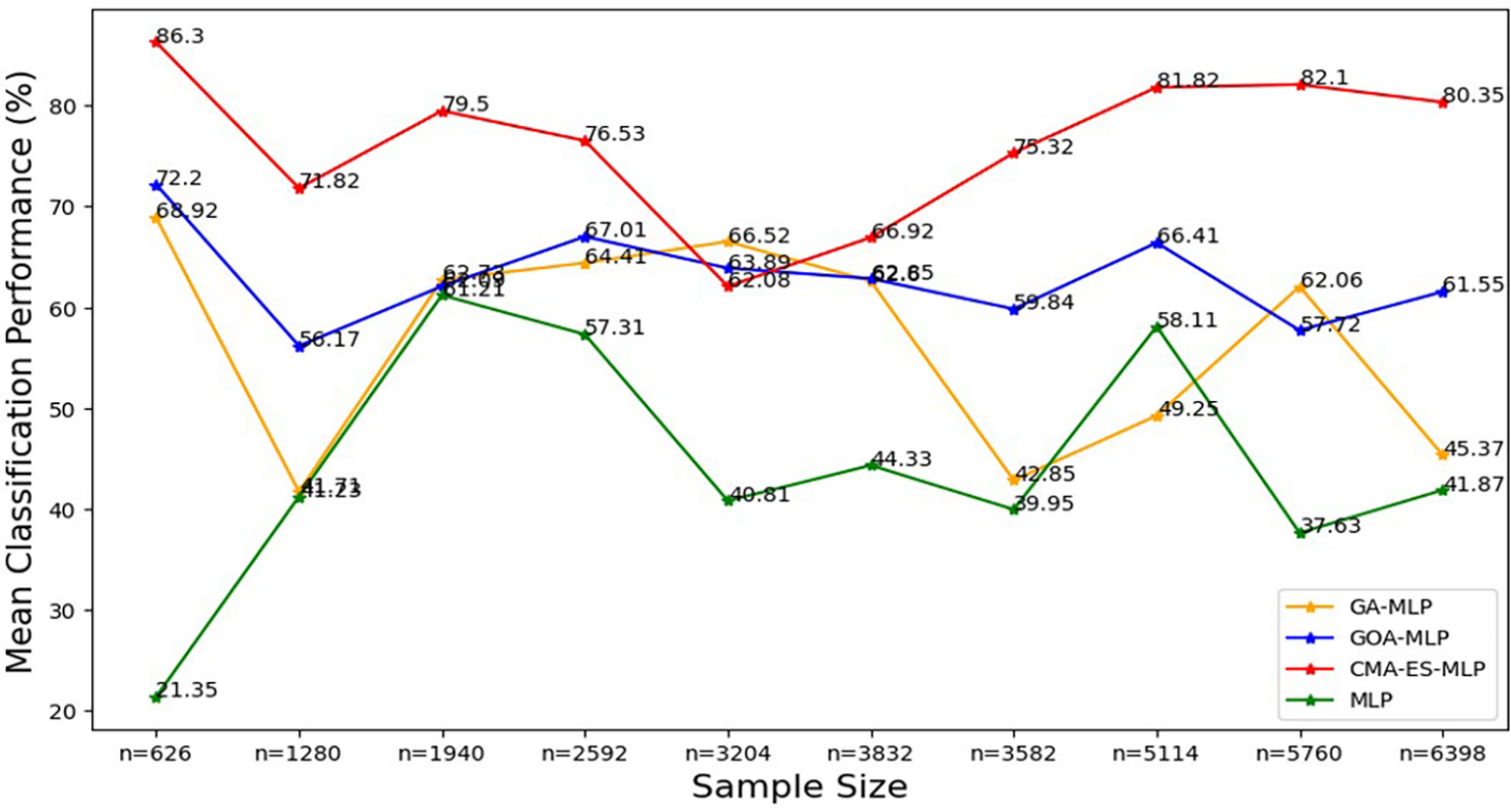
Mean of classification metrics for the basic MLP, GA-MLP, GOA-MLP and CMA-ES-MLP across the samples. The line graphs represent the performance of the basic MLP and each optimised MLP across the various sample sizes on average. This was to determine the impact of various sample sizes on the average performance of the models. The mean classification performance was computed by taking the average of overall classification accuracy, precision, sensitivity and specificity.

To ease the presentation and interpretation of the results, the results are presented by plotting each classification metric of all the ML classifiers under comparison across the sample sizes in Figures 2 to 8.

Figure 2 shows that, for the basic MLP classifier, the classification accuracy values fluctuate and do not follow a clear increasing or decreasing trend with different sample sizes. The GA-MLP shows that the classification accuracy values also fluctuate and do not exhibit a consistent pattern with the change in the sample size. For GOA-MLP, the classification accuracy values show fluctuations but are more stable than those of its competitors. For CMA-ES-MLP, the classification accuracy values appear to fluctuate with a significant drop for the 60% sample (n = 3832), but in most of the sample sizes (10% (n = 626), 20% (n = 1280), 40% (n = 2592), 70% (n = 3582), 80% (n = 5114), 90% (n = 5114), and 100% (n = 6398)), this classifier has the highest overall classification accuracy rates; that is, the ability to classify both subscribers and non-subscribers from the datasets. The precision rates for all classifiers across sample sizes are shown in Figure 3.

Figure 3 shows that the precision values for all the models fluctuated as the sample sizes increased and did not show a consistent upward or downward pattern as the sample size increased. Generally, relatively high rates of precision are shown for the CMA-ES-MLP in most of the sample sizes (10% (n = 626), 20% (n = 1280), 30% (n = 1940), 40% (n = 2592), 50% (n = 3204), 60% (n = 3832), 70% (n = 3582), 80% (n = 5114), and 100% (n = 6398)). This implies that CMA-ES-MLP has the highest ability to correctly classify positive cases (subscribers) out of all predicted positives compared to GA-MLP, GOA-MLP, and the basic MLP in most instances. The sensitivity/recall rates for all classifiers across sample sizes are presented in Figure 4.

Figure 4 shows a sharp increase from the smallest sample size (10% (n = 626)) to the second-smallest sample size (20% (n = 1280)) in the sensitivity/recall rate for the basic MLP. Thereafter, a steady increase was observed until n = 2592, followed by fluctuating values of sensitivity in the remaining sample sizes. Generally, the basic MLP with no optimization yielded the lowest sensitivity rates across all sample sizes compared to its competitors (except for the full dataset (n = 6398)). The second lowest sensitivity/recall rates were observed for GA-MLP across all samples, except for the 70% sample (n = 3582) and the 80% sample (n = 5114), so generally GA-MLP is the second worst performer among all four models. Figure 4 shows that the sensitivity/recall values for GOA-MLP and CMA-ES-MLP also show fluctuations but are generally relatively higher than those of the basic MLP and GA-MLP for most samples.

The sensitivity/recall rates for CMA-ES-MLP decreased slowly as the sample size increased (except when n = 5760). In general, the sensitivity rates for GA-MLP, GOA-MLP, and CMA-ES-MLP are more stable across the sample sizes relative to those derived from the basic MLP without optimization because they do not fluctuate rapidly, as in the case of the basic MLP. In most instances, GA-MLP and CMA-ES-MLP correctly classified the negatives (non-subscribers) better than GOA-MLP and basic MLP. The specificity rates for all classifiers across sample sizes are shown in Figure 5.

Figure 5 shows that for the basic MLP, the specificity values are relatively low and fluctuate with different sample sizes. The specificity for GA-MLP was highest for the smallest sample size (10% (n = 626)), followed by an upward trend between the second-smallest sample (20% (n = 1280)) and the fifth-lowest or sixth highest (50% (n = 3204)). Thereafter, it fluctuates, but for most sample sizes, its values are greater than those of the basic MLP and lower than those of the GOA-MLP. The specificity values for CMA-ES-MLP generally increased from the sixth highest sample (50% (3204)) to the full dataset (n = 6398) as the sample size increased. Generally, the CMA-ES-MLP classifies the positives (subscribers) correctly more accurately than the basic MLP, GA-MLP, and GOA-MLP. The F-measure rates for all classifiers across sample sizes are shown in Figure 6.

Figure 6 shows that for the basic MLP, the F-measure appears to fluctuate with different sample sizes without forming a clear upward or downward trend, and the basic MLP yielded the lowest F-measure across all sample sizes. For GA-MLP, generally, there seems to be an increase in the F-measure as the sample size increases from 20% (n=1280) to 50% (n=3240) and from 60% (3832) to 90% (5760); however, GA-MLP is the second worst performer in terms of the F-measure. Figure 6 also shows that the F-measure for the GOA-MLP fluctuates, and there is a significant drop in its performance for the whole dataset (n=1940); however, this classifier is generally the second-best performer in terms of the F-measure, after the CMA-ES-MLP. The execution times for all classifiers across sample sizes are shown in Figure 7.

Figure 7 shows that the basic MLP was the fastest to train, followed by CMA-ES-MLP. For the GA-MLP and GOA-MLP algorithms, there was an increasing trend whereby, as the sample sizes increased, the execution time also increased for these classifiers, but GA-MLP was the most expensive model when the sample size was at least 5114. The means of the classification metrics for all classifiers across the sample sizes are shown in Figure 8.

Figure 8 shows that the CMA-ES-MLP algorithm consistently achieved the highest mean accuracy across different sample sizes (except for the 50% sample size (n=3204)), indicating that it is the most accurate model overall. The GA-MLP and GOA-MLP algorithms showed varied performance, but for most sample sizes (10% (n=626), 20% (n=1280), 30% (n=1940), 40% (n=2592), 70% (n=3582), 80% (n=5114), and 100% (n=6398)), GA-GOA-MLP provided more accurate classifications than GA-MLP. The basic MLP algorithm consistently achieved the lowest mean classification accuracy, indicating its poor performance compared to its optimized variates. In general, the classifiers can be ranked in descending order of mean classification accuracy: CMA-ES-MLP, GOA-MLP, GA-MLP, and basic MLP.

## 1.6 Conclusion

This study was conducted to determine the impact of sample size on the classification ability and efficiency of GA, GOA, and CMA-CS, which are optimization algorithms for the MLP. The comparison was performed using line graphs of precision, F-measure, accuracy, sensitivity/recall, specificity, and execution time for basic MLP, GA-MLP, GOA-MLP, and CMA-ES-MLP across the ten samples. The line charts did not reveal a defined relationship between the performance of the classifiers across the sample sizes because the plots varied rapidly as the sample size increased. However, the execution time showed a clearer pattern as the sample size increased. The results revealed that GOA-MLP had more stable classification accuracy values than its competitors. Generally, the sensitivity rates for GA-MLP, GOA-MLP, and CMA-ES-MLP were more stable across the sample sizes relative to those derived from the basic MLP without optimization, since they did not fluctuate rapidly like those of the basic MLP.

The researchers concluded that the CMA-ES-MLP is the best model for this study in general because it maintains high rates of classification accuracy, F-measure, precision, and specificity for most sample sizes, and was the second-best performing classifier execution time. Furthermore, the mean classification metric results revealed that the CMA-ES-MLP algorithm consistently achieved the highest mean accuracy across nine different sample sizes, indicating that it is the most accurate model overall. The CMA-ES-MLP optimizer was identified as the most efficient optimization algorithm for an optimum MLP, as it was generally the most accurate optimizer, and it provided a lower execution time than GA-MLP and GOA-MLP, which did not increase noticeably as the sample size increased, implying that the CMA-ES optimizer is the most efficient optimizer for an optimum MLP compared with GA and GOA across all samples.

Generally, the sample size affects the performance of the MLP because the values of the classification metrics do not remain constant as the sample size changes. However, the results revealed that the values of the accuracy metrics for all the models fluctuated as the sample size increased, and there was no consistent increase or decrease in the classification performance of the algorithms as the sample size increased. On the other hand, the execution times for the GA and GOA optimizers increased as the sample size increased, but the execution time of the basic MLP remained the lowest and was almost constant as the sample size increased. Although CMA-ES had the lowest execution time compared to GOA and GA, it increased slightly when the sample size was at least 5114.

## Contribution

This study compared the performance of the basic MLP to MLPs optimized using GA, GOA, and CMA-ES, which has not been done in other studies; therefore, this is a contribution to the literature on MLP and optimization algorithms. Through this study, it is now known that the performance of MLP, GA-MLP, CMA-ES-MLP, GO-MLP, and GOA-MLP varies rapidly across the sample sizes, so we cannot generalize that the larger the sample size, the better the model, or vice versa. This novel knowledge extends the literature on ML classifiers, especially MLP. From the execution time results, the change in sample sizes revealed that the basic MLP was the fastest, followed by the CMA-ES-MLP, whereas in the other models, as the sample size increased, the execution time also increased. This implies that the CMA-ES-MLP is not just the most accurate, but also less expensive and has proven to be more stable in terms of training time as the sample size increases. This implies that the training time for the CMA-ES-MLP is least affected by the change in the datasets and using it with large datasets is likely not to affect its training time significantly as opposed to the GA and GOA. These results contribute novel knowledge about the efficiency of CMA-ES in optimizing the MLP.

The findings of this study also showed that training the MLP and its optimized variates on different samples that are randomly drawn from a larger dataset may aid in identifying the sample that can yield the most accurate classifier, as opposed to training the classifiers using one training dataset. More specifically, the selected model CMA-ES-MLP yielded the highest accuracy (overall classification accuracy, precision, and specificity) when the sample size was 5114, which is less than that of the mother dataset of 6398 observations. The best CMA-ES-MLP identified in this study competes well with classifiers that were the best performers from previous studies using the same dataset. For example, the best CMA-ES-MLP that was identified as the performer in this study has a classification accuracy of 90.18%, which is higher than that of the Meta-cost MLP (77.48%), ^29^ RF (86.08%), ^41^ and DT (87.5%). ^30^ This comparison does not ignore the fact that in some previous studies, the setting was different from that used in our study. It is recommended that a future study using the classifiers that were identified as the best from previous studies in Table 2 and the CMA-ES-MLP from this study be conducted to compare these classifiers under the same setting. The recommendations drawn from this study contribute new possible areas of research around ML classifiers, and the implications of the findings from this study contribute to a novel, accurate, and efficient approach to predicting the likelihood of a potential client subscribing to a term deposit using CMA-ES-MLP.

## Ethical considerations

This paper was written using parts of a PhD study whose proposal was presented at the school colloquium, where it received approval. It was subsequently submitted to the School Scientific Committee for approval as well. Then the proposal approved by the North-West University’s Faculty of Economic and Management Sciences Research Scientific Committee (FEMS-REC) on 30 June 2023, with the study classified as minimal risk. The ethics approval number is NWU-00684-22-A4.

## Data Availability

The data used in this study is a secondary dataset on direct marketing campaigns of a Portuguese banking institution named “Bank Marketing.” The dataset was obtained from the UCI Machine Learning Repository by the Center for Machine Learning and Intelligent Systems. The primary contributor for the data is. ^28^ The dataset can be accessed through https://archive.ics.uci.edu/ml/datasets/Bank+Marketing. DOI: 10.24432/C5K306. The researchers took some random samples to mimic different sample sizes so that they can successfully achieve the objective of study which is to determine the impact of sample size on the performance of optimisation algorithms for the MLP used in the prediction of client subscription to a term deposit. The dataset is licensed under CC BY 4.0 license which allows for its sharing and adaptation for any purpose (which imply that research purposes is included) provided that the appropriate credit is given (which is done in this paper in section 1.3.1).
