# Supplementary material for: Impact of sample size on optimisation algorithms for the MLP used in the prediction of client subscription to a term deposit
Source: F1000Res. 2026 Mar 2;14:1426. Originally published 2025 Dec 22. [Version 3] doi: 10.12688/f1000research.168092.3 (PMC12910200; doi:10.12688/f1000research.168092.3)
Supplement: Peer review history

# Reviewer report: Reviewer response for version 3

**DOI:** 10.5256/f1000research.197119.r463888

**Published:** 2026-03-17

**Authors:** Mohsen Mohammadagha

**Affiliations:** 1 University of Texas at Arlington, Arlington, Texas, USA

Overall assessment

 The paper has improved compared with earlier versions because it now includes a Kruskal–Wallis analysis and a short future-research section, but important consistency, reproducibility, and writing issues still remain. In its current form, I would lean toward “Approved with Reservations” rather than “Approved.”

 1- In Section 1.3.1, the authors say the raw sample sizes are 452, 904, 1356, 1808, 2261, 2713, 3165, 3617, 4069, and 4521, but in later tables and figures the “n” values become 626, 1280, 1940, 2592, 3204, 3832, 3582, 5114, 5760, and 6398, which are clearly SMOTE-balanced training sizes. This distinction must be made explicit every time sample size is mentioned.

 2- The abstract says “CMA-ES-MLP with a training sample of 5 114 was our ideal classifier”, but a casual reader may assume 5,114 is the original study sample, even though the original dataset size is only 4,521 and 5,114 appears to be a post-SMOTE balanced training size. The wording is misleading and should be corrected.

 3- The contribution section says the best model occurred at “the mother dataset of 6398 observations”, but the paper earlier states the original dataset has 4,521 observations. The 6,398 value appears to be the balanced training size, not the mother dataset size. This is a consistency error.

 4- Counting of samples is inconsistent: The methods say “nine (9) random samples” were drawn, but the listed sizes are 10%, 20%, 30%, 40%, 50%, 60%, 70%, 80%, 90%, and 100%, which is ten sample sizes, not nine.

 5- Statistical comparison is still not fully sufficient for the strength of the claims: The authors added the Kruskal–Wallis test, which is a welcome improvement, but the manuscript still makes strong claims such as “CMA-ES-MLP is the best model” without reporting repeated-run uncertainty, confidence intervals, or appropriate post-hoc pairwise comparisons. The result remains partly descriptive rather than fully inferential. If the purpose is to compare multiple ML methods across multiple conditions, a Friedman test with post-hoc comparisons (or another repeated-measures comparison framework) would usually align more closely with the comparative nature of the experiment than a simple Kruskal–Wallis presentation of mean ranks alone. At minimum, the authors should tone down the definitiveness of the conclusion. The manuscript does not clearly state whether results for each sample size come from one random draw / one split or from multiple repeated runs with different seeds. Without repeated runs, performance fluctuations may simply reflect sampling randomness rather than systematic sample-size effects.

 6- Reproducibility is still incomplete: The paper says the code is available on request, but the manuscript itself still lacks a clear, fully reproducible protocol: random seeds, exact optimizer settings, stopping criteria, hardware, timing protocol, and detailed hyperparameter-search settings should be reported in the paper or supplement, not only in code that may be requested later. The train/test procedure is described as 80/20, and SMOTE is said to be applied to the training data, which is good, but the details remain incomplete. The authors should specify: whether the test set was always held out before SMOTE, whether the same test set structure was used across all sample sizes, which SMOTE parameters were used, and whether features were scaled/normalized before training the MLP and optimizers.

 7- Comparison to prior studies is overstated: The manuscript compares its best classifier to results from prior studies on the same dataset and notes higher accuracy values, but the authors also acknowledge that settings differed. Because preprocessing, train/test split, balancing strategy, features, and evaluation protocols may vary widely, these cross-study comparisons should be presented more cautiously as informal context, not as direct benchmarking, as in Benchmark papers (e.g., original Moro et al., 2014) report modest accuracies (~88-90% on large versions with simple models like logistic regression; ML models vary but rarely exceed 92-93% without heavy tuning due to imbalance and noise).

 8- Sample-size and results inconsistencies: In the Figure 2 discussion, the paper says “90% (n = 5114)”, but 5,114 corresponds to the 80% sample’s balanced training size, not the 90% sample. The 90% balanced training size is 5,760 according to Table 4.

 9- In the Figure 6 discussion, the text says “50% (n=3240)”, but Table 4 shows the 50% balanced training size as 3,204, not 3,240.

 10- In the same Figure 6 discussion, the text says there is a drop for the “whole dataset (n=1940)”, but 1,940 corresponds to the 30% balanced training size, not the whole dataset. This is an error. The whole-dataset balanced training size is listed as 6,398.

 11- Table 3 contains a row with “n = 2026” on the unbalanced side, which does not fit the stated 10%-increment sampling scheme and appears inconsistent with the progression of training sample sizes. This row needs to be checked and corrected.

 12- The paragraph interpreting Table 7 lists only a subset of sample sizes (n = 1446, 1809, 2170, 2026, 2894, 3255 and 3617) and omits others, while also mixing different notions of sample size. The authors should either list all sample sizes consistently or avoid listing them at all in that sentence.

 13- The conclusion says the study was conducted on “GA, GOA, and CMA-CS”; “CMA-CS” is a typo and should be “CMA-ES.”

 14- The manuscript says the model is best for predicting “credit card subscription”, but the entire paper is about term deposit subscription. This is a substantive wording error, not merely stylistic.

 15- Some statements about stability are still too qualitative, such as saying one model is “more stable” without defining stability quantitatively. If stability is important, the authors should report something like standard deviation across repeated runs or explicitly state that “stability” is being judged only visually from line plots.

 16- In the abstract, “multilayer perception (MLP)” should be “multilayer perceptron (MLP).”

 17- Citation Format Issues: The manuscript contains improper in-text citation formatting that creates grammatically incomplete sentences. For example: "According to,14 there are several..." is missing the author name(s) before the superscript citation "in the study by,15,16 and it has been..." similarly omits the required author name(s). Corrections needed entire manuscript:  Use narrative citations, for example: “According to Abdel-Basset et al. [14], there are several optimization algorithms. I provide a reference demonstrating an example for narrative citations and statistical evaluation in machine learning research. (Reference 1) “Mohammad Agha, M., Asadi, S., & Naeini, H. K. (2025). Evaluating machine learning performance using Python for neural network models in urban transportation in New York City case study. Journal of Economy and Technology.”

 18- Grammar and sentence-level edits: “This imply” → “This implies”

 19- “which imply” → “which implies” (when the subject is singular)

 20- “Tables 7 presents” → “Table 7 presents” or “Tables 5–7 present,” depending on intent

 21- “The sample size do not” → “The sample sizes do not” or “Sample size does not”

 22- The recommendations section has several grammatical issues, for example: “Only simple random sampling with replacement was performed” conflicts with the earlier use of "stratified sampling" wording.

 23- “ca compare” should be “can compare.”

 24- “ADYSN” should likely be “ADASYN.”

 25- “results form this study” should be “results from this study.”

 26- One sentence says “Table shows the hyperparameter estimates…” but should specify “Table 4 shows…” for clarity.

Is the work clearly and accurately presented and does it cite the current literature?

Partly

If applicable, is the statistical analysis and its interpretation appropriate?

Partly

Are all the source data underlying the results available to ensure full reproducibility?

Yes

Is the study design appropriate and is the work technically sound?

Yes

Are the conclusions drawn adequately supported by the results?

Partly

Are sufficient details of methods and analysis provided to allow replication by others?

No

Reviewer Expertise:

Civil Engineering and Computer Science

I confirm that I have read this submission and believe that I have an appropriate level of expertise to confirm that it is of an acceptable scientific standard, however I have significant reservations, as outlined above.

# Reviewer report: Reviewer response for version 3

**DOI:** 10.5256/f1000research.197119.r463885

**Published:** 2026-03-17

**Authors:** Kamal Shah

**Affiliations:** 1 Prince Sultan University, Riyadh, Riyadh Province, Saudi Arabia

Accepted

Is the work clearly and accurately presented and does it cite the current literature?

Yes

If applicable, is the statistical analysis and its interpretation appropriate?

Yes

Are all the source data underlying the results available to ensure full reproducibility?

Yes

Is the study design appropriate and is the work technically sound?

Yes

Are the conclusions drawn adequately supported by the results?

Yes

Are sufficient details of methods and analysis provided to allow replication by others?

Yes

Reviewer Expertise:

Applied mathematics

I confirm that I have read this submission and believe that I have an appropriate level of expertise to confirm that it is of an acceptable scientific standard.

# Reviewer report: Reviewer response for version 2

**DOI:** 10.5256/f1000research.195714.r458634

**Published:** 2026-02-19

**Authors:** Venkata Mohit Tamanampudi

**Affiliations:** 1 JP Morgan Chase, New York City, USA

This is a well-structured and carefully conducted study that provides a clear comparison of GA, GOA, and CMA-ES optimization algorithms for improving MLP performance under varying sample sizes. The methodology is detailed, the evaluation metrics are comprehensive, and the graphical analysis helps illustrate how classification performance fluctuates as the dataset size changes. The conclusions are generally well supported by the reported results, particularly the identification of CMA-ES-MLP as a strong and computationally efficient performer.

 If the authors want to further improve the paper, they could consider:

- Expanding the discussion on why classification metrics fluctuate across sample sizes, providing deeper analytical insights rather than primarily descriptive observations.
- Including statistical significance testing to strengthen the validity of the performance comparisons between models.
- Evaluating the models on additional datasets to improve generalizability beyond the single Bank Marketing dataset.
- Providing more detailed information on hyperparameter tuning and computational settings to enhance reproducibility.
- Discussing the potential impact of SMOTE and oversampling on model bias and real-world deployment scenarios.

Is the work clearly and accurately presented and does it cite the current literature?

Yes

If applicable, is the statistical analysis and its interpretation appropriate?

Yes

Are all the source data underlying the results available to ensure full reproducibility?

Yes

Is the study design appropriate and is the work technically sound?

Yes

Are the conclusions drawn adequately supported by the results?

Yes

Are sufficient details of methods and analysis provided to allow replication by others?

Yes

Reviewer Expertise:

Artificial Intelligence, deep learning, LLM's, Machine learning, NLP, Devops, AI Agents

I confirm that I have read this submission and believe that I have an appropriate level of expertise to confirm that it is of an acceptable scientific standard.

# Response

**Published:** 2026-02-24

**Authors:** Tlhalitshi Montshiwa

**Affiliations:** Business Statistics, North West University Faculty of Economic and Management Sciences, Mahikeng, North West, South Africa

Thank you for your constructive feedback. We will use it to improve this work and our future works in general.

# Reviewer report: Reviewer response for version 2

**DOI:** 10.5256/f1000research.195714.r458631

**Published:** 2026-02-16

**Authors:** Kamal Shah

**Affiliations:** 1 Prince Sultan University, Riyadh, Riyadh Province, Saudi Arabia

Authors have presented Impact of sample size on optimisation algorithms for the MLP used in the prediction of client subscription to a term deposit. Results seems good applicable. I recommend after revision like: 

 The presented work is good applicable. I recommend after revision like:

1. Improve abstract
2. Improve and update your paper literature with more sophisticated results on mentioned diseases recently published.
3. Include the importance of fractional derivatives now a days a popular tool of research.
4. Check that all figures/tables have been cited.
5. References must be double check as now a days some works have retracted so that to avoid retracted article citations.
6. Revise conclusion.
7. Run spell check .

Include recent references of 2025/2024 like:

 Reference 1 *Heliyon* 10.3 (2024).

 Reference 2 *Heliyon* 10.13 (2024).

 Reference 3 *Results in Applied Mathematics* 21 (2024): 100420.

Is the work clearly and accurately presented and does it cite the current literature?

Yes

If applicable, is the statistical analysis and its interpretation appropriate?

Yes

Are all the source data underlying the results available to ensure full reproducibility?

Yes

Is the study design appropriate and is the work technically sound?

Yes

Are the conclusions drawn adequately supported by the results?

Yes

Are sufficient details of methods and analysis provided to allow replication by others?

Yes

Reviewer Expertise:

Applied mathematics

I confirm that I have read this submission and believe that I have an appropriate level of expertise to confirm that it is of an acceptable scientific standard, however I have significant reservations, as outlined above.

## References

[1] : Statistical and computational analysis for corruption and poverty model using Caputo-type fractional differential equations. *Heliyon* .2024;10(3) : 10.1016/j.heliyon.2024.e25440 10.1016/j.heliyon.2024.e25440 PMC1084795038327401

[2] : Analysis of nonlinear Burgers equation with time fractional Atangana-Baleanu-Caputo derivative. *Heliyon* .2024;10(13) : 10.1016/j.heliyon.2024.e33842 10.1016/j.heliyon.2024.e33842 PMC1126987439055819

[3] : Numerical modelling of advection diffusion equation using Chebyshev spectral collocation method and Laplace transform. *Results in Applied Mathematics* .2024;21: 10.1016/j.rinam.2023.100420 10.1016/j.rinam.2023.100420

# Response

**Published:** 2026-02-24

**Authors:** Tlhalitshi Montshiwa

**Affiliations:** Business Statistics, North West University Faculty of Economic and Management Sciences, Mahikeng, North West, South Africa

Thank you for your feedback. We will consider it when we modify the paper.

# Reviewer report: Reviewer response for version 2

**DOI:** 10.5256/f1000research.195714.r458632

**Published:** 2026-02-16

**Authors:** Joseph Chukwunweike

**Affiliations:** 1 Gist Limited, Bristol, UK

**1. Background & framing**

  The opening motivation about MLP “parameter growth” is relevant, but tighten the claim by specifying what “parameters” means (weights, biases) and why this matters for generalization and training cost. Define “spatial information” or remove it, because the task is tabular bank marketing and not image-like inputs. When stating MLP is “most-used,” add one or two concrete citations and avoid vague superlatives. Several sentences break grammar due to citation placement (e.g., “According to,14 …”); rewrite using narrative form with author names. The scope paragraph should explicitly justify why GA/GOA/CMA-ES were selected over PSO/DE besides “scarce literature,” and state the hypothesis: sample size changes may differentially affect optimizer stability and runtime. Clarify “efficiency” early as a multi-criteria notion (accuracy metrics + execution time), and note potential trade-offs. End the section with contributions bullet list to guide the reader. Keep terminology consistent throughout the paper overall.

**2. Related work**

  Table 2 is useful, but the narrative around it should separate (i) prior work on the UCI Bank Marketing dataset and (ii) prior work on metaheuristic optimization of MLPs. Where you say “results vary depending on setting,” add specific examples (different feature sets, resampling, train/test splits). Several references are cited without a subject (“including.28–30”); add author names and verbs. Consider adding a short paragraph summarizing 2023–2025 developments in MLP optimization and evaluation, even if the core study is historical, to address recency concerns. Clarify what is novel: not merely comparing optimizers, but evaluating sensitivity to sample size under consistent conditions. Also, avoid implying that “optimized MLPs are always best”; phrase it as an observed trend in surveyed studies. Finally, define acronyms at first use (BOA, BPSO) and ensure the reader can map each to a cited source. Briefly note reporting norms for compute and runtime.

**3. Methods & dataset**

  The dataset description is clear (UCI Bank Marketing, 4,521 rows), but the sampling paragraph contains inconsistencies: you report n=452 at 10%, yet later figures mention n=626 and other totals exceeding 4,521. Reconcile all sample sizes and explain whether SMOTE changes the effective training n. “Nine samples” is stated, but ten sizes (10%–100%) are listed; correct the count. Stratified sampling with replacement should specify the seed and how many replications were run per sample size. One-hot encoding to 42 features is plausible; list which variables were expanded and confirm handling of “unknown” levels. If SMOTE is applied, state whether it is applied only to training folds to prevent leakage, and specify the SMOTE parameters. Provide MLP architecture (layers, activations), optimizer settings, and hyperparameter search bounds for GA/GOA/CMA-ES so the work is reproducible. Also describe missing-value handling and any scaling/normalization steps.

**4. Evaluation metrics & experimental protocol**

  The metric definitions (accuracy, specificity, precision, recall, F-measure, execution time) are standard, but the presentation would improve by stating the primary metric(s) used for model selection and why. Because the outcome is imbalanced (term deposit subscription), consider adding balanced accuracy or PR-AUC as a supplementary metric, or justify why not. The protocol should state the train/test split ratio for each sample size, and whether cross-validation is used. If you generate one test set per sample size, confirm it is held out before SMOTE. Execution time should be measured on fixed hardware; report CPU, RAM, and software versions. The paper makes comparative claims (“best model”) without uncertainty; add confidence intervals via repeated runs, and apply a nonparametric test (e.g., Friedman across methods with post-hoc) as suggested in the open review. Also clarify whether timing includes optimizer search plus MLP training, or final fit.

**5. Results & figures**

  Figures 2–8 communicate trends well, but ensure captions include the exact sample sizes (currently some captions and text disagree). When describing fluctuations, quantify them (min/max accuracy per model) and add a table of mean±std across repeated runs. The “significant drop” at the 60% sample for CMA-ES-MLP needs explanation check random sampling variance, class balance, or convergence settings. The narrative sometimes switches between classifying positives vs negatives; keep terminology consistent with the metric being discussed (precision vs specificity). For Figure 7, clarify units (seconds) and whether timing was averaged. The text says GOA-MLP is “more stable” but does not define stability; consider a simple stability score (standard deviation across replications). Figure 8 averages multiple metrics; explain the aggregation choice and whether metrics were normalized before averaging to avoid scale bias. Finally, add one summary table of best-at-each-sample-size results to improve readability at a glance.

**6. Conclusion, contribution & reproducibility**

  The conclusion summarizes findings clearly, but correct the typo “CMA-CS” to “CMA-ES.” Avoid stating “CMA-ES-MLP is the best” as a definitive fact without statistical testing; phrase it as “performed best under the reported protocol” and add uncertainty. The contribution section is promising; strengthen it by explicitly stating what practitioners can take away (e.g., CMA-ES’s runtime scalability vs GA/GOA). The data availability statement says code is available “upon request”; for reproducibility, provide a public repository or, at minimum, include pseudo-code and full hyperparameter settings in an appendix. Add details on random seeds, number of optimizer iterations, stopping criteria, and evaluation repeats, as highlighted by the reviewer report. Finally, add a short limitations paragraph: single dataset, single task, discrete sampling with replacement, and dependence on SMOTE choices. These are minor additions that materially improve clarity and defensibility. For readers and future replicators alike.

Is the work clearly and accurately presented and does it cite the current literature?

Partly

If applicable, is the statistical analysis and its interpretation appropriate?

Yes

Are all the source data underlying the results available to ensure full reproducibility?

Yes

Is the study design appropriate and is the work technically sound?

Yes

Are the conclusions drawn adequately supported by the results?

Yes

Are sufficient details of methods and analysis provided to allow replication by others?

Yes

Reviewer Expertise:

Automation, Process Control, Image processing, pattern Recognition

I confirm that I have read this submission and believe that I have an appropriate level of expertise to confirm that it is of an acceptable scientific standard.

# Reviewer report: Reviewer response for version 2

**DOI:** 10.5256/f1000research.195714.r453926

**Published:** 2026-02-02

**Authors:** Mohsen Mohammadagha

**Affiliations:** 1 University of Texas at Arlington, Arlington, Texas, USA

**Literature update**: While I understand the historical context, adding a brief section on 2023-2025 developments would strengthen rather than compromise the framework.

**Citation Format Issues:** The manuscript contains improper in-text citation formatting that creates grammatically incomplete sentences. 

 For example: "According to,14 there are several..." is missing the author name(s) before the superscript citation "in the study by,15,16 and it has been..." similarly omits the required author name(s). 

 Corrections needed entire manuscript:  Use narrative citations, 

 For example: “According to Abdel-Basset et al. [14], there are several optimization algorithms. 

 I provide a reference demonstrating an example for narrative citations and statistical evaluation in machine learning research. (Reference 1)

**Statistical rigor is insufficient: **I appreciate your acknowledgment of this issue. However, statistical comparison is not an optional enhancement—it is a fundamental requirement for comparing ML algorithms. The experiments have already been conducted; calculating confidence intervals and applying appropriate statistical tests (e.g., Friedman with post‑hoc analyses) would require limited additional effort and is essential for scientific validity. Without such analyses, claims about which algorithm is “best” remain descriptive rather than statistically substantiated. Since statistical tests were not included, the conclusion that "CMA-ES-MLP is the best model" is technically a subjective opinion, not a scientific fact. Without p-values or confidence intervals, the differences seen in Figures 2–8 could easily be due to random chance.

**Technical Typos in Core Definitions:** In the Conclusion (Section 1.6), they refer to the algorithm as **"CMA-CS"** rather than **"CMA-ES"**.

**This revision is largely unchanged:** Version 2 appears almost identical to Version 1, with a minor addition (“code available upon request”).

 In my view, addressing this point would substantially improve the quality and credibility of the work.

Is the work clearly and accurately presented and does it cite the current literature?

Partly

If applicable, is the statistical analysis and its interpretation appropriate?

Partly

Are all the source data underlying the results available to ensure full reproducibility?

Yes

Is the study design appropriate and is the work technically sound?

Yes

Are the conclusions drawn adequately supported by the results?

Partly

Are sufficient details of methods and analysis provided to allow replication by others?

No

Reviewer Expertise:

Civil Engineering and Computer Science

I confirm that I have read this submission and believe that I have an appropriate level of expertise to confirm that it is of an acceptable scientific standard, however I have significant reservations, as outlined above.

## References

[1] : Evaluating machine learning performance using python for neural network models in urban transportation in New York city case study. *Journal of Economy and Technology* .2026;4: 10.1016/j.ject.2025.11.001 266-283 10.1016/j.ject.2025.11.001

# Reviewer report: Reviewer response for version 1

**DOI:** 10.5256/f1000research.185252.r446950

**Published:** 2026-01-10

**Authors:** Mohsen Mohammadagha

**Affiliations:** 1 University of Texas at Arlington, Arlington, Texas, USA

This study compares the performance of three optimization algorithms—Genetic Algorithm (GA), Grasshopper Optimization Algorithm (GOA), and Covariance Matrix Adaptation Evolution Strategy (CMA-ES)—for optimizing Multilayer Perceptron (MLP) neural networks across varying sample sizes. Using a Portuguese banking dataset (4,521 observations) from the UCI repository, the researchers evaluated how sample size affects classification performance when predicting client subscription to term deposits. The study employed SMOTE for data balancing and tested ten different sample sizes (10%-100%). Results identified CMA-ES-MLP as the best overall performer with high accuracy, precision, and specificity, while maintaining competitive execution time.

**1. Is the work clearly and accurately presented and does it cite current literature?**

 Recommendations: Update literature review with 2023-2025 papers.

**2. Is the study design appropriate and is the work technically sound? Yes**

**3. Are sufficient details of methods and analysis provided to allow replication by others?**

 Recommended **: **Code availability​, Random seeds​, Incomplete hyperparameter specifications​, Software versions​, MLP architecture.

**4. If applicable, is the statistical analysis and its interpretation appropriate?**

 It can be suggested to conduct runs with statistical tests (e.g., Friedman or any other methods) and report confidence intervals.

**5. Are all the source data underlying the results available to ensure full reproducibility? **Yes

**6. Are the conclusions drawn adequately supported by the results?**

 It is recommended to add confidence intervals, statistical tests (e.g., McNemar's test, Friedman test with post-hoc analysis)

**Citation Format Issues:** The manuscript contains improper in-text citation formatting that creates grammatically incomplete sentences. Specifically:

- "According to,14 there are several..." is missing the author name(s) before the superscript citation
- "in the study by,15,16 and it has been..." similarly omits the required author name(s)

**Corrections needed entire manuscript:**

 Use narrative citations, for example: “According to Abdel-Basset et al. [14], there are several optimization algorithms.

 I provide a reference demonstrating an example for statistical evaluation in machine learning research.

 (Reference 1)

Is the work clearly and accurately presented and does it cite the current literature?

Partly

If applicable, is the statistical analysis and its interpretation appropriate?

Partly

Are all the source data underlying the results available to ensure full reproducibility?

Yes

Is the study design appropriate and is the work technically sound?

Yes

Are the conclusions drawn adequately supported by the results?

Partly

Are sufficient details of methods and analysis provided to allow replication by others?

No

Reviewer Expertise:

Civil Engineering and Computer Science

I confirm that I have read this submission and believe that I have an appropriate level of expertise to confirm that it is of an acceptable scientific standard, however I have significant reservations, as outlined above.

## References

[1] : Evaluating machine learning performance using python for neural network models in urban transportation in New York city case study. *Journal of Economy and Technology* .2026;4: 10.1016/j.ject.2025.11.001 266-283 10.1016/j.ject.2025.11.001

# Response

**Published:** 2026-01-23

**Authors:** Tlhalitshi Montshiwa

**Affiliations:** Business Statistics, North West University Faculty of Economic and Management Sciences, Mahikeng, North West, South Africa

**Comment 1:** Update literature review with 2023-2025 papers.

**Response 1:** Thank you for the comment. Kindly note that this article is derived from a PhD study conducted in 2020, which informed the initial scope and selection of the literature. Removal of references below 2023 may lead to misalignment with the theoretical framework and objectives of the study.

**Comment 2:** Code availability​, Random seeds​, Incomplete hyperparameter specifications​, Software versions​, MLP architecture.

**Response 2:** Thank you for this comment. The codes utilised for the analysis are available upon request. These codes include detailed information on the random seed settings, hyperparameter specifications, software versions, and the MLP architecture used in the study. This statement is included in the manuscript in data availability section.

**Comment 3:** It can be suggested to conduct runs with statistical tests (e.g., Friedman or any other methods) and report confidence intervals.

**Response 3:** This is a valuable suggestion, and the inclusion of confidence intervals as well as statistical comparison tests such as the Friedman and McNemar tests would enhance the robustness of the analysis. Due to the limited scope and objectives of the current study, these analyses were not implemented at this stage. However, they will be incorporated in future work to provide a more comprehensive statistical comparison of the models.

**Comment 4:** It is recommended to add confidence intervals, statistical tests (e.g., McNemar's test, Friedman test with post-hoc analysis)

**Response 4:** This is a valuable suggestion, and the inclusion of confidence intervals and formal statistical comparison tests such as McNemar’s and Friedman tests would indeed strengthen the robustness of the analysis. However, given the limited scope and objectives of the current study, the focus was placed on comparative predictive performance using standard evaluation metrics. Incorporating additional inferential statistical tests is therefore left as a potential extension for future work.
